# Supplementary material for: Global motion processing in infants’ visual cortex and the emergence of autism
Source: Commun Biol. 2023 Mar 28;6:339. doi: 10.1038/s42003-023-04707-3 (PMC10050234; doi:10.1038/s42003-023-04707-3)
Supplement: Supplementary file 2 — Supplementary Information [file 42003_2023_4707_MOESM2_ESM.pdf]

Supplementary Information

Included as items of supplementary information are the following:

Supplementary Figure 1

Comparison of topographies across the four experimental conditions.

Supplementary Figure 2

Detail plots of EEG activation over the 9 visual cortical Areas of Interest (AOIs) from 473 infants in the Normative Sample.

Supplementary Figure 3

Difference in attention to stimulus between included and rejected (excluded) infants for the analysis.

Supplementary Figure 4

Normality test of Laterality Scores in the four conditions.

Supplementary Table 1

Results of statistical tests and models presented in the main text and in Supplementary Information.

Supplementary Table 2

Data attrition due to analysis-level exclusions.

Supplementary Table 3

Descriptive statistics of participants from the two studies at the initial EEG session at 5 months (final samples).

Supplementary Table 4

Descriptive statistics of participants from the EASE study included in analyses with ADOS-2 CS (EASE sub-samples).

Supplementary Table 5

Providing measures of data quality.

Supplementary Notes 1

Outlining inclusion and exclusion criteria for participant recruitment in both EASE and BATSS studies.

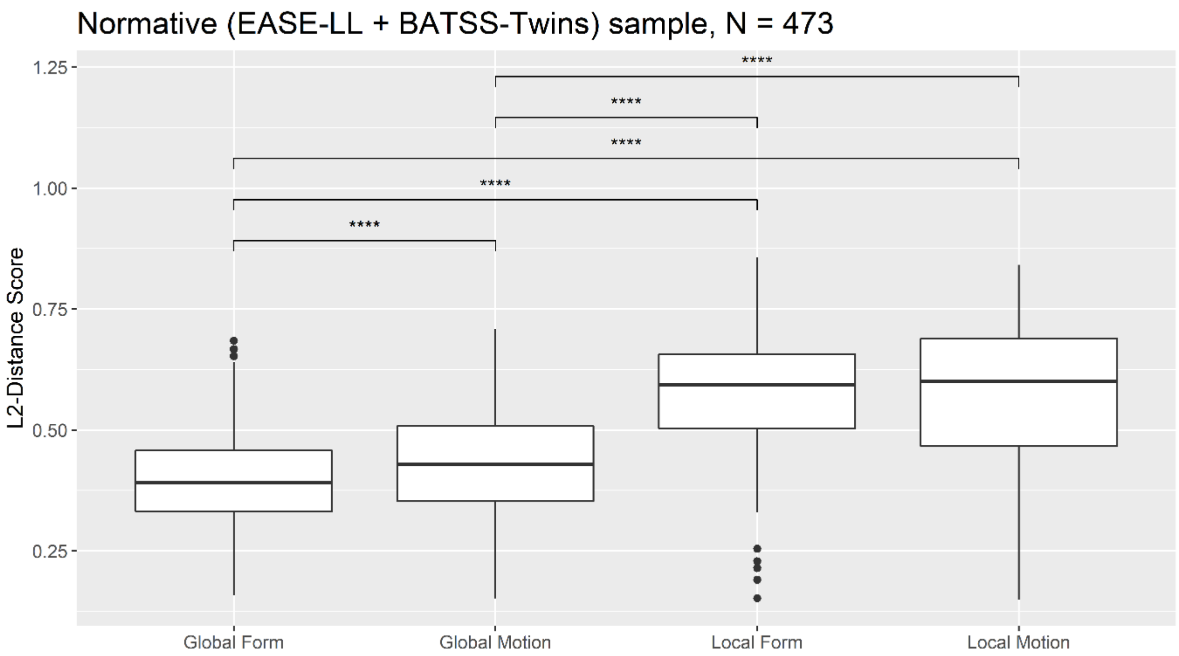

Supplementary Figure 1 | Comparison of topographies across the four experimental conditions. We calculated an L2 (Euclidean) distance from each individual topographical profile-vector to the grand average activation vector (a flat vector) over all conditions, all AOIs, and all participants, and compared the distributions of these distance scores between the four conditions. The mean distance of each global condition was found to be different from all the other three conditions (Global Form: all ***$p < .0001$, Global Motion: all ***$p < .0001$), signifying their distinctive activation topographies and implying recruitment of different neural networks in both processes. The mean distances between the two local change conditions were found to be not significantly different ($p = .78$). Boxplots show the sample median, and the first and third quartiles; whiskers show minimum and maximum ($\pm$1.5 s.d.); dots are outliers ($\pm$1.96 s.d.). See **Supplementary Table 1** for complete statistics.

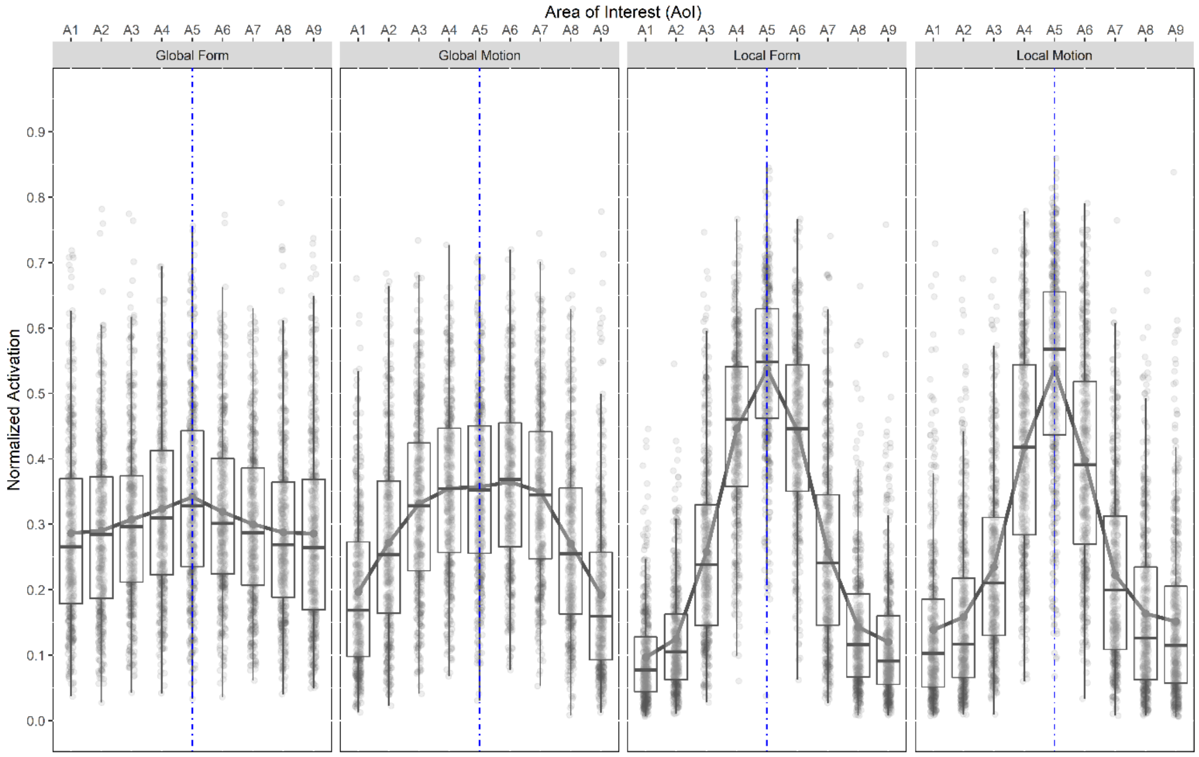

Supplementary Figure 2 | Detail plots of EEG activation over the 9 visual cortical Areas of Interest (AOIs) from 473 infants in the Normative Sample. Here, we provided a more detailed look to the visual cortical EEG activation patterns across the nine AOIs from the EASE LL (*n* = 21) and BATSS (*n* = 452) infants making up the Normative Sample group, shown in **Fig. 2c** (main text). To provide a more comprehensive (yet still concise) descriptive statistics of these EEG patterns, data are plotted as mean (bold dots connected by lines), median, first and third quartiles, minimum, and maximum (boxplots and whiskers, respectively), as well as the individual data points (small shaded dots). Non-overlapping mean and median in an AOI indicates a somewhat skewed distribution.

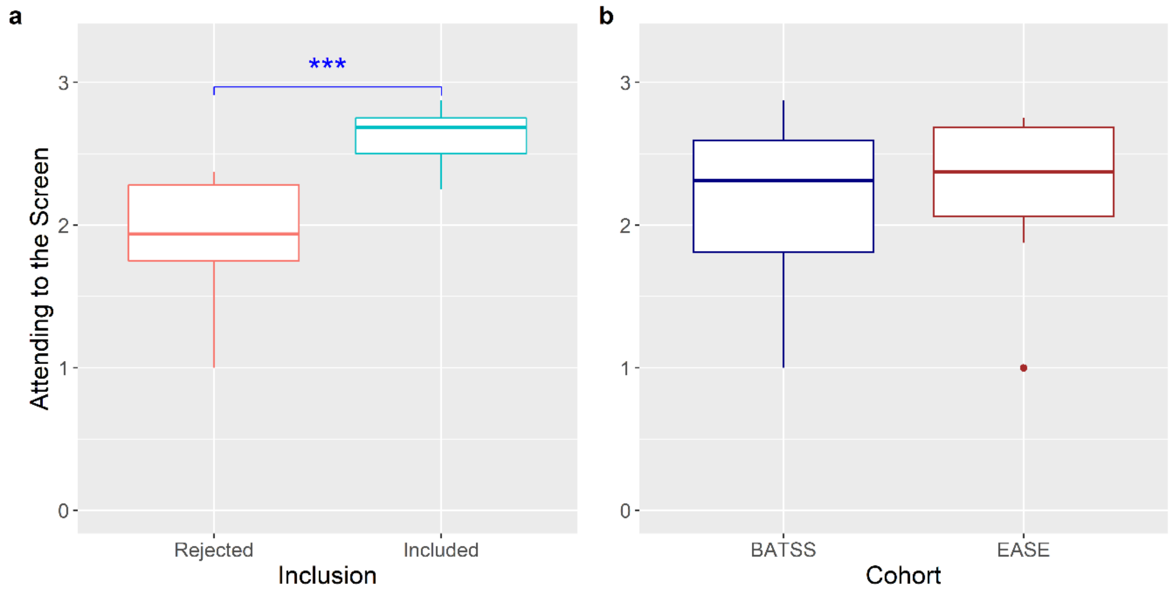

Supplementary Figure 3 | Difference in attention to stimulus between included and rejected (excluded) infants for the analysis. A. As expected infants (n = 10, random selection, EASE/BATSS ratio = 50%/50%) from the Included category (see **Methods**) had a higher (***$p < .001$) proportion of time looking at the screen compared to those in the Rejected category ($n = 10$, random selection, EASE/BATSS ratio = 50%/50%). **B.** The two samples had equal looking time to the screen ($p > .50$, same infants as in a). Boxplots show the sample median, and the first and third quartiles; whiskers show minimum and maximum ($\pm$1.5 s.d.); dots are outliers ($\pm$1.96 s.d.). See **Supplementary Table 1** for complete statistics.

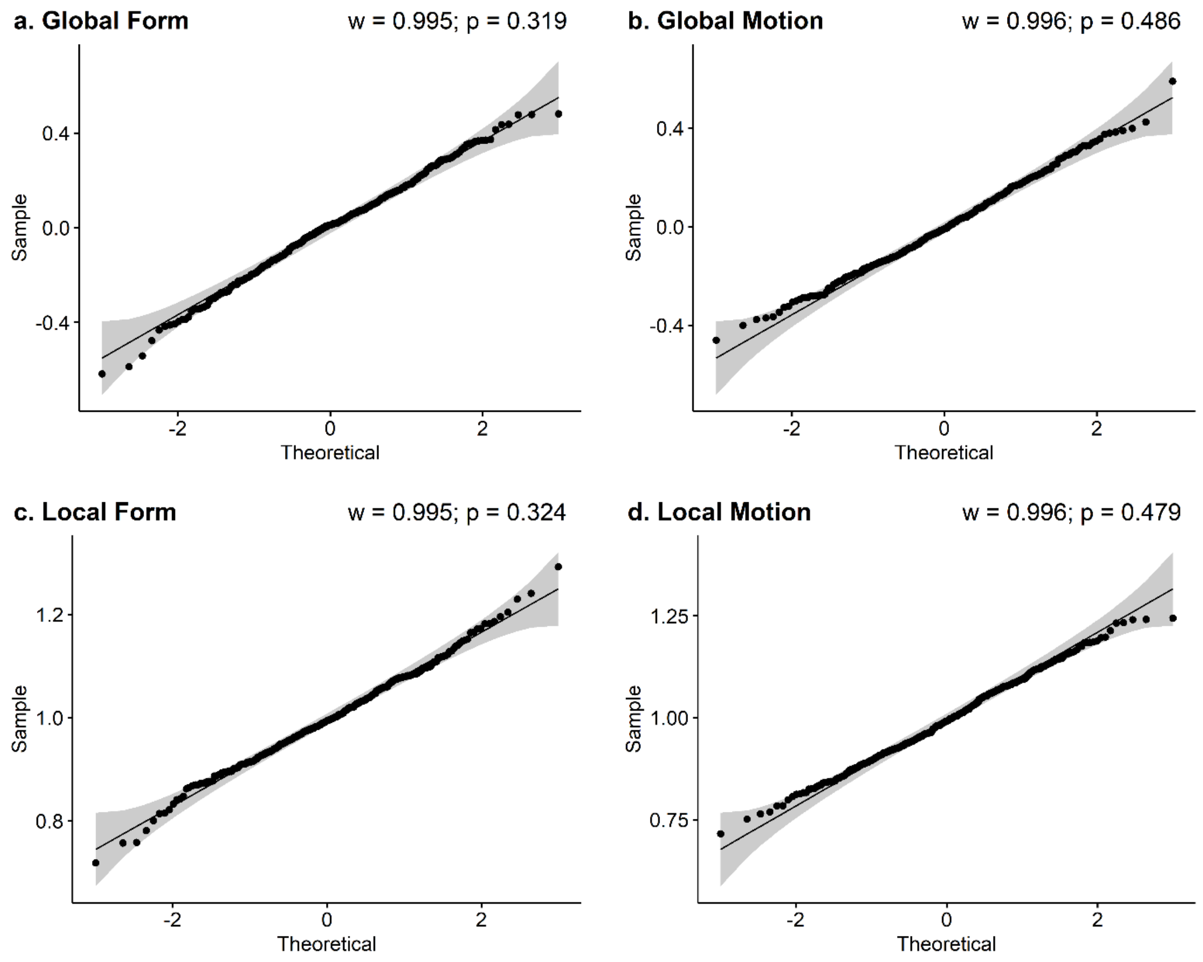

Supplementary Figure 4 | Normality test of Laterality Scores in the four conditions. a. and **b.** The normality of residuals (after regressing out sex and age) of Laterality Scores in both Global Form and Motion conditions was satisfied (Shapiro-Wilk, both $p > .25$). **c.** and **d.** In both local change (Local Form and Motion) conditions, the score distributions were slightly skewed and were corrected using a square-root transformation on the scores. The resulting distributions did not divert from normality ($p > .25$). Subsequent model fitting for both local change conditions were done on these transformed scores. In all plots, test statistic and p-value are presented on the upper-right corner. See also **Supplementary Table 1**.

Supplementary Table 1 | Results of statistical tests and models presented in the main text and in Supplementary Information

All analyses presented in the manuscript were performed using standard functions/scripts in R language. All *p*-values are <u>two-tailed</u> unless otherwise specified.

***** GFo = Global Form, GMo = Global Motion, LFo = Local Form, LMo = Local Motion; EL = Elevated Likelihood for ASD; EL11 = Elevated Likelihood, 11 participants who did not continue to partake in ADOS-2 at 36 months; npar = number of parameters

Category	Section in Main Text	Referred to in Display Item or on Manus. Page	Test / Model	Parameter estimates	*N*	*p*
Main result	*Association between lateralized activation and later autistic symptoms in the EASE sample*	Figs. 2a, 3a	Bivariate correlation (Pearson's *r*)	*r*(GMo x ADOS2_36mo, all) = .465<br>*r*(GMo x ADOS2_36mo, EL) = .448	55<br>39	< .001<br>.004
Main result	*Association between lateralized activation and later autistic symptoms in the EASE sample*	Figs. 2a, 3a	OLS regression [ADOS-2 CS total scores at 36 months], model with only main effects + effect sizes (partial η^2)	<u>Model coefficient | effect size:</u><br>β(sex_Male) = .90 | part. η^2 = .070<br>β(age_36mo) = .001 | part. η^2 = .0004<br>β(group_LL) = -.85 | part. η^2 = .052<br>β(GFo) = 1.90 | part. η^2 = .027<br>β(GMo) = 5.01 | part. η^2 = .170<br>β(LFo) = -1.59 | part. η^2 = .010<br>β(LMo) = 1.35 | part. η^2 = .016	55	 .075<br>.895<br>.128<br>.272<br>.004<br>.515<br>.408
Main result	*Association between lateralized activation and later autistic symptoms in the EASE sample*	Figs. 2a, 3a	OLS regression [ADOS-2 CS total scores at 36 months], model with interaction effects (moderation) of GMo + effect sizes (partial η^2)	<u>Model coefficient | effect size:</u><br>β(sex_Male) = .77 | part. η^2 = .072<br>β(age_36mo) = -.002 | part. η^2 = .0005<br>β(group_LL) = -1.18 | part. η^2 = .053<br>β(GMo) = 7.22 | part. η^2 = .179<br>β(GMo*group_LL) = -6.42 | p. η^2 = .019<br>β(GMo*sex_Male) = -3.56 | p. η^2 = .011<br>β(group_LL*sex_Male) = .57 | p. η^2 = .007<br>β(GMo*group_LL*sex_Male) = 5.63<br>| p. η^2 = .014	55	 .209<br>.882<br>.154<br>.005<br>.242<br>.322<br>.615<br>.432
Main result	*Association between lateralized activation and later autistic symptoms in the EASE sample*	Fig. 2a, inset	Bivariate correlation; right-tailed *p*-value (Pearson's *r*)	*r*(GMo x ADOS2_24mo, EL11) = .596	11	.027
Main result	*Association between lateralized activation and later autistic symptoms in the EASE sample*	Fig. 2b	Linear mixed model & estimated marginal means (EMM) effect sizes (Cohen's d)	<u>*Only GMo*</u><br>EL hi-ADOS[i] – EL lo-ADOS[ii]: *d* = .25<br>EL hi-ADOS[i] – LL controls[iii]: *d* =.33<br>EL hi-ADOS[i] – Twins[iv]: *d* = .31<br>EL lo-ADOS[ii] – LL controls[iii]: *d* = .10<br>EL lo-ADOS[ii] – Twins[iv]: *d* = .001<br>LL controls[iii] – Twins[iv]: *d* = -.12	14[i]<br>25[ii]<br>16[iii]<br>452[iv]	 .029<br>.002<br>.003<br>.680<br>.999<br>.515

<table>
<tr><th></th><th></th><th></th><th></th><th></th><th></th><th></th></tr>
<tr><td>Main result</td><td>Association between lateralized activation and later autistic symptoms in the EASE sample</td><td>Figs. 3b-d</td><td>Linear mixed model & estimated marginal means (EMM) effect sizes (Cohen’s d)</td><td><u>GFo</u>
EL hi-ADOS[i)] – EL lo-ADOS[ii)]: d = -.02
EL hi-ADOS[i)] – LL controls[iii)]: d = .11
EL hi-ADOS[i)] – Twins[iv)]: d = .03
EL lo-ADOS[ii)] – LL controls[iii)]: d = .15
EL lo-ADOS[ii)] – Twins[iv)]: d = .08
LL controls[iii)] – Twins[iv)]: d = -.13

<u>LMo</u>
EL hi-ADOS – EL lo-ADOS: d = -.01
EL hi-ADOS – LL controls: d = -.05
EL hi-ADOS – Twins: d = -.06
EL lo-ADOS – LL controls: d = -.04
EL lo-ADOS – Twins: d = -.06
LL controls – Twins: d = -.001

<u>LFo</u>
EL hi-ADOS – EL lo-ADOS: d = .002
EL hi-ADOS – LL controls: d = .06
EL hi-ADOS – Twins: d = .006
EL lo-ADOS – LL controls: d = .06
EL lo-ADOS – Twins: d = .005
LL controls – Twins: d = -.08</td><td>14[i)]
25[ii)]
16[iii)]
452[iv)]</td><td>
.996
.581
.982
.305
.822
.434

.998
.943
.889
.967
.906
1.000

.999
.921
.999
.889
1.000
.805</td></tr>
<tr><td>Main result</td><td>Association between lateralized activation and later autistic symptoms in the EASE sample</td><td>Fig. 2c, Supplementary Fig. 2</td><td>Linear mixed models + robust expected marginal means (EMM) contrast analysis [Normative Sample[i)] – EL high-ADOS[ii)] in 9 AOIs] with FDR / Benjamini-Hochberg correction + effect sizes (Cohen’s d)</td><td><u>GFo (contrast | effect size)</u>
M(A1) = -.061 | d = -.05
M(A2) = -.017 | d = -.01
M(A3) = .035 | d = .03
M(A4) = .075 | d = .06
M(A5) = .035 | d = .03
M(A6) = -.021 | d = -.02
M(A7) = -.004 | d = -.003
M(A8) = -.033 | d = -.03
M(A9) = -.017 | d = -.01

<u>GMo (contrast | effect size)</u>
M(A1) = -.120 | d = -.10
M(A2) = -.095 | d = -.08
M(A3) = -.030 | d = -.03
M(A4) = .085 | d = .07
M(A5) = .100 | d = .08
M(A6) = .031 | d = .03
M(A7) = .027 | d = .02
M(A8) = .058 | d = .05
M(A9) = .026 | d = .02

<u>LFo (contrast | effect size)</u>
M(A1) = .001 | d = .001
M(A2) = -.015 | d = -.01
M(A3) = -.035 | d = -.03
M(A4) = -.060 | d = -.06
M(A5) = -.004 | d = -.004
M(A6) = .042 | d = .04
M(A7) = .023 | d = .02
M(A8) = -.010 | d = -.009
M(A9) = .008 | d = .008

<u>LMo (contrast | effect size)</u>
M(A1) = .032 | d = .03
M(A2) = .031 | d = .02
M(A3) = -.020 | d = -.02
M(A4) = -.082 | d = -.06</td><td>473[i)]
14[ii)]</td><td>
.411
.729
.647
.349
.647
.729
.919
.647
.729

.009
.026
.469
.044
.026
.469
.469
.202
.469

.963
.963
.804
.533
.963
.804
.963
.963
.963

.622
.622
.678
.296</td></tr>
</table>

<table>
<tr><td></td><td></td><td></td><td></td><td>M(A5) = -.038 | d = -.03
M(A6) = .048 | d = .04
M(A7) = .032 | d = .03
M(A8) = .007 | d = .006
M(A9) = .024 | d = .02</td><td></td><td>.622
.622
.622
.851
.678</td></tr>
<tr><td>Main result</td><td>Twin modelling</td><td>p. 9</td><td>Bivariate Correlation, across-phenotypes (Pearson’s r)</td><td>r(GFo x GMo) = .025
r(GFo x LFo) = .031
r(GFo x LMo) = .016
r(GMo x LFo) = .099
r(GMo x LMo) = .045
r(LFo x LMo) = .257</td><td>366</td><td>.631
.552
.758
.049
.345
< .0001</td></tr>
<tr><td>Main result</td><td>Twin modelling</td><td>p. 9</td><td>Bivariate Correlation, intra-zygosity/ICC (Pearson’s r)</td><td>GFo: r(MZ) = -.085 | r(DZ) = -.145
GMo: r(MZ) = .307 | r(DZ) = -.049
LFo: r(MZ) = -.121 | r(DZ) = .148
LMo: r(MZ) = .086 | r(DZ) = -.051</td><td>366</td><td>.406 | .182
.002 | .656
.239 | .175
.400 | .640</td></tr>
<tr><td>Main result</td><td>Twin modelling</td><td>p. 9</td><td>Likelihood ratio (LR) test & AE model for GMo</td><td><u>LR test:</u>
ADE model: -2LL = -269.46; npar = 4
ACE model: -2LL = -267.61; npar = 4
AE model: -2LL = -267.61; npar = 3
E model: -2LL = -261.50; npar = 2
ADE vs AE: ΔLL = 1.85 (Δdf = 1)
ADE vs E: ΔLL = 7.96 (Δdf = 2)
ACE vs AE: ΔLL = 0.00 (Δdf = 1)
ACE vs E: ΔLL = 6.11 (Δdf = 2)

<u>AE model:</u>
A: .226 [.047, .392]
E: .774 [.608, .953]
h^2 = A + D = .226</td><td>366</td><td>

.174
.019
1.000
.047</td></tr>
<tr><td>Main result</td><td>Twin modelling</td><td>p. 9</td><td>Likelihood ratio (LR) test & E model for GFo</td><td><u>LR test:</u>
ACE model: -2LL = -176.08; npar = 4
AE model: -2LL = -176.08; npar = 3
E model: -2LL = -176.08; npar = 2
ACE vs AE: ΔLL = 0.00 (Δdf = 1)
ACE vs E: ΔLL = 0.00 (Δdf = 2)

<u>E model:</u>
E: 1.000 [1.000, 1.000]
h^2 = A + D = .000 (no heritability)</td><td>366</td><td>

1.000
1.000</td></tr>
<tr><td>Main result</td><td>Twin modelling</td><td>p. 9</td><td>Likelihood ratio (LR) test & AE model for LMo</td><td><u>LR test:</u>
ACE model: -2LL = -653.27; npar = 4
AE model: -2LL = -653.27; npar = 3
E model: -2LL = -653.27; npar = 2
ACE vs AE: ΔLL = 0.00 (Δdf = 1)
ACE vs E: ΔLL = 0.00 (Δdf = 2)

<u>E model:</u>
E: 1.000 [1.000, 1.000]
h^2 = A + D = .000 (no heritability)</td><td>366</td><td>

1.000
1.000</td></tr>
<tr><td>Main result</td><td>Twin modelling</td><td>p. 9</td><td>Likelihood ratio (LR) test & AE model for LFo</td><td><u>LR test:</u>
ACE model: -2LL = -778.12; npar = 4
AE model: -2LL = -778.11; npar = 3
E model: -2LL = -778.11; npar = 2
ACE vs AE: ΔLL = 0.01 (Δdf = 1)</td><td>366</td><td>

.911</td></tr>
</table>

				# ACE vs E: ΔLL = 0.01 (Δdf = 2)<br><br><u>E model:</u><br>E: 1.000 [1.000, 1.000]<br>h^2 = A + D = .000 (no heritability)		.994
Main result	*Association with autistic traits in the BATSS sample*	pp. 9-10	Bivariate correlation (Pearson's *r*) [Laterality Score vs. ITC, QCHAT]	<u>ITC total scores @14 months:</u><br>*r*(GMo x ITC_total) = -.085<br>*r*(GFo x ITC_total) = -.029<br>*r*(LMo x ITC_total) = -.092<br>*r*(LFo x ITC_total) = -.143<br><br><u>QCHAT scores @24 months:</u><br>*r*(GMo x QCHAT) = -.059<br>*r*(GFo x QCHAT) = -.174<br>*r*(LMo x QCHAT) = .064<br>*r*(LFo x QCHAT) = -.002	274<br><br><br><br><br><br>204	 .160<br>.632<br>.128<br>.018<br><br><br>.402<br>.013<br>.359<br>.975
Main result	*Association with autistic traits in the BATSS sample*	pp. 9-10	GEE [ITC total scores],<br>model with only main effects + effect sizes (partial η^2)	<u>Model coefficient | effect size:</u><br>*β*(sex_Male) = -2.08 | part. η^2 = .013<br>*β*(age_14mo) = .05 | part. η^2 = .008<br>*β*(GMo) = -3.56 | part. η^2 = .007<br>*β*(GFo) = -0.84 | part. η^2 = .0005<br>*β*(LMo) = -2.38 | part. η^2 = .005<br>*β*(LFo) = -4.78 | part. η^2 = .011	274	 .054<br>.154<br>.158<br>.716<br>.257<br>.090
Main result	*Association with autistic traits in the BATSS sample*	pp. 9-10	GEE [QCHAT scores],<br>model with only main effects + effect sizes (partial η^2)	<u>Model coefficient | effect size:</u><br>*β*(sex_Male) = 3.79 | part. η^2 = .045<br>*β*(age_24mo) = -.005 | part. η^2 = .0002<br>*β*(GMo) = -1.72 | part. η^2 = .001<br>*β*(GFo) = -5.52 | part. η^2 = .038<br>*β*(LMo) = 1.56 | part. η^2 = .002<br>*β*(LFo) = -.56 | part. η^2 = .0002	204	 .002<br>.857<br>.604<br>.006<br>.546<br>.856
Main result	*Association with autistic traits in the BATSS sample*	pp. 9-10	OLS regression [ITC total scores, QCHAT scores], models to check explanatory power of 4 Laterality Scores	<u>OLS regression of ITC total scores:</u><br>*F*(4, 269) = 2.033<br>R^2 = .015<br><br><u>OLS regression of QCHAT scores:</u><br>*F*(4, 199) = 1.856<br>R^2 = .017	274<br><br><br><br>204	 *p* = .090<br><br><br><br>*p* = .120
Additional result	*Association between lateralized activation and later autistic symptoms in the EASE sample*	pp. 8-9	OLS regression [ADOS-2 CS total scores at 24 months],<br>model with only main effects + effect sizes (partial η^2)	<u>Model coefficient | effect size:</u><br>*β*(sex_Male) = .03 | part. η^2 = ~.000<br>*β*(age_24mo) = .003 | part. η^2 = .002<br>*β*(group_LL) = -1.40 | part. η^2 = .086<br>*β*(GFo) = -.52 | part. η^2 = .002<br>*β*(GMo) = 3.49 | part. η^2 = .063<br>*β*(LFo) = -.84 | part. η^2 = .002<br>*β*(LMo) = 3.02 | part. η^2 = .052	68	 .950<br>.729<br>.020<br>.738<br>.049<br>.736<br>.075
Additional result	*Association between lateralized activation and later autistic symptoms in the EASE sample*	pp. 8-9	OLS regression [ADOS-2 CS total scores at 24 months],<br>model with interaction effects (moderation) of GMo + effect sizes (partial η^2)	<u>Model coefficient | effect size:</u><br>*β*(sex_Male) = .30 | part. η^2 = ~.000<br>*β*(age_24mo) = -.0001 | part. η^2 = ~.000<br>*β*(group_LL) = -1.96 | part. η^2 = .084<br>*β*(GMo) = 5.75 | part. η^2 = .077<br>*β*(GMo*group_LL) = -6.16 | part. η^2 = ~.00<br>*β*(GMo*sex_Male) = -4.00 | part. η^2 = .004<br>*β*(group_LL*sex_Male) = 1.19<br>| part. η^2 = .016<br>*β*(GMo*group_LL*sex_Male) = 10.20	68	 .629<br>.995<br>.020<br>.030<br>.276<br>.300<br>.329<br><br>.194

				| part. η^2 = .028		
Additional result	*Discussion*	Fig. 4a	Contrast analysis [Normative Sample[i)] – EL high-ADOS[ii)] in 9 AOIs] with BH/FDR correction (only the significant AOIs) + effect sizes (Cohen's d)	<u>*GFo* (only effect size)</u><br>No significant AOI: all *d* < | .05| <br><u>*GMo* (only effect size)</u><br>No significant AOI: all *d* < | .03| <br><u>*LFo* (only effect size)</u><br>No significant AOI: all *d* < | .03| <br><u>*LMo* (only effect size)</u><br>No significant AOI: all *d* < | .03|	473[i)]<br>14[ii)]	all *p* > .50<br><br>all *p* > .50<br><br>all *p* > .50<br><br>all *p* > .50
Additional result	*Discussion*	p. 11	Bivariate correlation (Pearson's *r*) [GMo Laterality Score vs. age at EEG meas.]	EASE: *r*(GMo x age_5mo) = .028<br>BATSS: *r*(GMo x age_5mo) = -.072	73<br>452	.814<br>.126
Additional result	*Discussion*	Fig. 4b	Contrast analysis [Normative Sample[i)] – EL high-ADOS[ii)] in 9 flipped AOIs] with BH/FDR correction (only the significant fAOIs) + effect sizes (Cohen's d)	<u>*GMo* (contrast | effect size)</u><br>*M*(A4) = .137 | *d* = .12<br>*M*(A5) = .100 | *d* = .09<br>*M*(A9) = -.084 | *d* = -.07	473[i)]<br>14[ii)]	< .001<br>.018<br>.048
Additional result	*Methods: Association analyses with autistic traits and symptoms*	p. 20	OLS regression (ADOS-2 SA sub-scores at 36 months), model with only main effects + effect sizes (partial η^2)	<u>Model coefficient | effect size:</u><br>β(sex_Male) = 1.25 | part. η^2 = .105<br>β(age_36mo) = .0003 | part. η^2 = ~.000<br>β(group_LL) = -.76 | part. η^2 = .034<br>β(GFo) = 2.24 | part. η^2 = .031<br>β(GMo) = 4.64 | part. η^2 = .125<br>β(LFo) = -.27 | part. η^2 = ~.000<br>β(LMo) = 1.23 | part. η^2 = .011	55	.028<br>.979<br>.217<br>.242<br>.016<br>.920<br>.498
Additional result	*Methods: Association analyses with autistic traits and symptoms*	p. 20	OLS regression (ADOS-2 RRB sub-scores at 36 months), model with only main effects + effect sizes (partial η^2)	<u>Model coefficient | effect size:</u><br>β(sex_Male) = .22 | part. η^2 = .003<br>β(age_36mo) = .001 | part. η^2 = ~.000<br>β(group_LL) = -.38 | part. η^2 = .007<br>β(GFo) = 3.91 | part. η^2 = .067<br>β(GMo) = 3.33 | part. η^2 = .052<br>β(LFo) = -4.05 | part. η^2 = .037<br>β(LMo) = 2.90 | part. η^2 = .042	55	.728<br>.937<br>.594<br>.082<br>.129<br>.201<br>.171
Additional result	*Methods: Twin modelling of Laterality Scores*	p. 21	Tests of modelling assumptions: t-tests for equality of means and variances	<u>Within-Pair Equality of Mean:</u><br>GFo: -.064 < $\Delta\mu$ < .014<br>GMo: -.017 < $\Delta\mu$ < .052<br>LFo: -.057 < $\Delta\mu$ < .012<br>LMo: -.070 < $\Delta\mu$ < .012<br><br><u>Within-Pair Equality of Variance:</u><br>GFo: .84 < σ_1^2/σ_2^2 < .1.50<br>GMo: .71 < σ_1^2/σ_2^2 < 1.27<br>LFo: 1.12 < σ_1^2/σ_2^2 < 2.00<br>LMo: .65 < σ_1^2/σ_2^2 < 1.17	366	.212<br>.319<br>.203<br>.163<br><br>.449<br>.701<br>.007<br>.359

				<u>Across-Zygosity Equality of Mean:</u><br>GFo: $-.013 < \Delta\mu < .065$<br>GMo: $-.025 < \Delta\mu < .044$<br>LFo: $-.034 < \Delta\mu < .035$<br>LMo: $-.022 < \Delta\mu < .060$<br><br><u>Across-Zygosity Equality of Variance:</u><br>GFo: $.88 < \sigma_1^2/\sigma_2^2 < .1.58$<br>GMo: $.79 < \sigma_1^2/\sigma_2^2 < 1.42$<br>LFo: $.68 < \sigma_1^2/\sigma_2^2 < 1.22$<br>LMo: $.74 < \sigma_1^2/\sigma_2^2 < 1.33$		.192<br>.593<br>.987<br>.353<br><br><br>.263<br>.688<br>.553<br>.983
Supplementary information	*Scalp topographies in response to basic visual stimuli in normative infants*	Supplementary Fig. 1	Linear mixed model & estimated marginal means (EMM) effect sizes (Cohen's d)	GFo – GMo: *d* = -.23<br>GFo – LFo: *d* = -1.28<br>GFo – LMo: *d* = -1.23<br>GMo – LFo: *d* = -1.05<br>GMo – LMo: *d* = -1.00<br>LFo – LMo: *d* = .05	473	< .0001<br>< .0001<br>< .0001<br>< .0001<br>< .0001<br>.78
Supplementary information	*Methods: Infant-level & epoch-level exclusions of bad EEG data*	Supplementary Fig. 3	Wilcoxon tests [Attending to the screen]	Included – Rejected: *W* = 94<br>EASE – BATSS: *W* = 54.5	20	.001<br>.761
Supplementary information	*Methods: Twin modelling of Laterality Scores*	Supplementary Fig. 4	Tests of modelling assumptions: Shapiro-Wilks' tests of normality of phenotypes	GFo: w = .995<br>GMo: w = .996<br>$\sqrt{(LFo + 1)}$: w = .995<br>$\sqrt{(LMo + 1)}$: w = .996	366	.319<br>.486<br>.324<br>.479

***** GFo = Global Form, GMo = Global Motion, LFo = Local Form, LMo = Local Motion, SA = Social Affect, RRB = Restricted and Repetitive Behavior; LL = log-likelihood; npar = number of parameters

Supplementary Table 2 | Data attrition due to analysis-level exclusions

	EASE dataset	BATSS dataset
Initial sample size	92†	599‡
No EEG data	-1	-36
Sample having EEG	91	563
Exclusion due to bad EEG data	-18	-111
**Final sample**	**73**	**452**

†The same participants/cohort as in ref.[1]
‡From the 622 individual twins (311 twin-pairs) admitted to the study[2], 23 individuals were excluded due to specific medical conditions (i.e., twin-to-twin transfusion syndrome; TTTS, low birth weight, spina bifida, and seizures at birth)

<u>**Cohort-specific sub-samples**</u>

EASE

Final sample	73
Sub-sample with ADOS-2 CS at 24 mo.	68
Sub-sample with ADOS-2 CS at 36 mo.	55

BATSS

Final sample	452
Sub-sample with complete twin-pair	366
Sub-sample with ITC at 14 mo.	274
Sub-sample with QCHAT at 24 mo.	204

Supplementary Table 3 | Descriptive statistics of participants from the two studies at the initial EEG session at 5 months (final samples)

	EASE – EL	EASE – LL controls	BATSS – Twins
N	52	21	452
Female	25	13	222

Monozygotic (MZ)	n/a	n/a	242
Age in months (mean ± s.d.)	5.40 ± 0.56	5.36 ± 0.49	5.52 ± 0.29
MSEL* Early Learning Composite Scale	96.9 ± 11.1	101.1 ± 7.6	94.1 ± 9.7

*Mullen Scales of Early Learning (MSEL)[3]

Supplementary Table 4 | Descriptive statistics of participants from the EASE study included in analyses with ADOS-2 CS (EASE sub-samples)

ADOS-2 at 24 months	**EASE – EL**		**EASE – LL controls**
N	50		18
Female	24		10
Age in months (mean ± s.d.)	24.87 ± 0.99		25.01 ± 1.30
MSEL* Early Learning Composite Scale	96.5 ± 11.1		100.3 ± 7.2
ADOS-2 at 36 months	**EASE - EL high-ADOS**	**EASE - EL low-ADOS**	**EASE – LL controls**
N	14	25	16
Female	5	14	8
Age in months (mean ± s.d.)	36.98 ± 0.76	36.79 ± 0.91	36.52 ± 0.61
MSEL* Early Learning Composite Scale	97.4 ± 10.8	97.2 ± 11.0	100.4 ± 7.3

*Mullen Scales of Early Learning (MSEL)[3]

Supplementary Table 5 | Number of trials (epochs) of EEG data used in the analysis, broken down by group and stimulus modality (mean ± s.d.)

	EASE EL low-ADOS	EASE EL high-ADOS	EASE LL controls	BATSS Twins
Visual form	165.5 ± 41.0	154.0 ± 49.3	161.3 ± 39.4	143.2 ± 45.8
Visual motion	163.7 ± 40.2	152.1 ± 50.7	163.1 ± 40.0	145.7 ± 46.0

Supplementary Notes 1 | Information on Study Exclusion/Inclusion Criteria in EASE and BATSS

Early Autism in Sweden (EASE) Study

Elevated Likelihood (EL) for ASD

Inclusion

- Age between 4 months and 6 months 3 weeks OR younger than 11 months
- Older full sibling with ASD or ADHD (presence of community clinical diagnosis).
- Parent with ASD or ADHD (presence of community clinical diagnosis)
- At least one parent speaks testing language to child at home (does not need to be parent's native language)

Exclusion

- Diagnosis of epilepsy or history of fits/convulsions in infant (not including febrile convulsions)
- Known presence of genetic syndrome (in proband or infant) clearly related to ASD (e.g., TSC, FXS, 22q11, 16p11.2, Rett's)
- Presence of known significant uncorrected vision or hearing impairment in infant (reported to parent by a doctor or health professional)
- Infant was premature (pre 36 weeks)
- Infant is looked after by the state (e.g., foster care), or other situation in which neither birth parent is involved in the infant's care
- Presence of known significant developmental or medical condition in infant likely to affect brain development or infant's ability to participate in the study (e.g., Cerebral Palsy, Down's syndrome, cystic fibrosis, foetal alcohol syndrome)

Low Likelihood (LL) for ASD

Inclusion

- Age between 4 months and 6 months 3 weeks
- Older full sibling with typical development (by parent report)
- At least one parent speaks testing language to child at home (does not need to be parent's native language)

Exclusion

- Diagnosis of epilepsy or history of fits/convulsions in infant

- Known presence of genetic syndrome (in proband or infant) clearly related to ASD (e.g., TSC, FXS, 22q11, 16p11.2, Rett's)
- Presence of known significant uncorrected vision or hearing impairment in infant (reported to parent by a doctor or health professional)
- Infant was premature (pre 36 weeks)
- Infant is looked after by the state (e.g., foster care), or other situation in which neither birth parent is involved in the infant's care
- Presence of known significant developmental or medical condition in infant likely to affect brain development or infant's ability to participate in the study (e.g., Cerebral Palsy, Down's syndrome, cystic fibrosis)
- Parent has ASD-specific concerns about their infant
- Presence of ASD, ADHD or language disorder in 1 to 2nd degree relatives

In addition, LL infants should be recruited to sex-match the EL sample.

BabyTwins in Sweden (BATSS) Study

General Criteria (also described in ref[11])

Inclusion

- Same-sex twin of age 4 – 5 months
- The twins must hear Swedish at home from at least one parent and live with at least one biological parent
- Parents also needed to be willing to share information about medical and psychiatric history in the family, demographic background and delivery

Exclusion

- Hearing or vision impairments
- Premature birth (defined as prior to week 34 of gestation)
- Epilepsy or seizures
- Medical conditions that were likely to affect brain development
- Ability to participate in the study
- Presence of known genetic syndromes

References

1 Nyström, P., Jones, E., Darki, F., Bölte, S. & Falck-Ytter, T. Atypical topographical organization of global form and motion processing in 5-month-old infants at risk for Autism. *Journal of autism and developmental disorders* **51**, 364-370 (2021).

2 Falck-Ytter, T. *et al.* The Babytwins Study Sweden (BATSS): a multi-method infant twin study of genetic and environmental factors influencing infant brain and behavioral development. *Twin Research and Human Genetics* **24**, 217-227 (2021).

3 Mullen, E. M. *Mullen scales of early learning*. (AGS Circle Pines, MN, 1995).
