# Supplementary material for: Global motion processing in infants’ visual cortex and the emergence of autism
Source: Commun Biol. 2023 Mar 28;6:339. doi: 10.1038/s42003-023-04707-3 (PMC10050234; doi:10.1038/s42003-023-04707-3)
Supplement: Supplementary file 3 — Description of Additional Supplementary Files [file 42003_2023_4707_MOESM3_ESM.pdf]

Description of Additional Supplementary Files

File Name: Supplementary Video 1

Description: Experimental stimulus used for both Local and Global Form conditions. Provided as movie

clip in a separate .zip file.

File Name: Supplementary Video 2

Description: Experimental stimulus used for both Local and Global Motion conditions. Provided as a movie

clip in a separate .zip file.

File Name: Supplementary Data 1

Description: Source tables containing numeric data to reproduce all the figures in the main text and in the supplementary information. Provided as .csv tables in a separate .zip file.
