# Supplementary material for: Global motion processing in infants’ visual cortex and the emergence of autism
Source: Commun Biol. 2023 Mar 28;6:339. doi: 10.1038/s42003-023-04707-3 (PMC10050234; doi:10.1038/s42003-023-04707-3)
Supplement: Supplementary file 7 — Reporting Summary [file 42003_2023_4707_MOESM7_ESM.pdf]

nature portfolio

Corresponding author(s): Irzam Hardiansyah

Last updated by author(s): Mar 1, 2023

Reporting Summary

Nature Portfolio wishes to improve the reproducibility of the work that we publish. This form provides structure for consistency and transparency in reporting. For further information on Nature Portfolio policies, see our Editorial Policies and the Editorial Policy Checklist.

Statistics

For all statistical analyses, confirm that the following items are present in the figure legend, table legend, main text, or Methods section.

n/a	Confirmed	
☐	☒	The exact sample size (n) for each experimental group/condition, given as a discrete number and unit of measurement
☐	☒	A statement on whether measurements were taken from distinct samples or whether the same sample was measured repeatedly
☐	☒	The statistical test(s) used AND whether they are one- or two-sided<br>*Only common tests should be described solely by name; describe more complex techniques in the Methods section.*
☐	☒	A description of all covariates tested
☐	☒	A description of any assumptions or corrections, such as tests of normality and adjustment for multiple comparisons
☐	☒	A full description of the statistical parameters including central tendency (e.g. means) or other basic estimates (e.g. regression coefficient) AND variation (e.g. standard deviation) or associated estimates of uncertainty (e.g. confidence intervals)
☐	☒	For null hypothesis testing, the test statistic (e.g. F, t, r) with confidence intervals, effect sizes, degrees of freedom and P value noted<br>*Give P values as exact values whenever suitable.*
☐	☒	For Bayesian analysis, information on the choice of priors and Markov chain Monte Carlo settings
☐	☒	For hierarchical and complex designs, identification of the appropriate level for tests and full reporting of outcomes
☐	☒	Estimates of effect sizes (e.g. Cohen's d, Pearson's r), indicating how they were calculated

Our web collection on statistics for biologists contains articles on many of the points above.

Software and code

Policy information about availability of computer code

Data collection	EEG data collection was done using EGI/EEG system and MATLAB software; custom-made scripts available upon a reasonable request
Data analysis	All pre-processing and analysis works were done in R and MATLAB; custom-made scripts available upon a reasonable request

For manuscripts utilizing custom algorithms or software that are central to the research but not yet described in published literature, software must be made available to editors and reviewers. We strongly encourage code deposition in a community repository (e.g. GitHub). See the Nature Portfolio guidelines for submitting code & software for further information.

Data

Policy information about availability of data

All manuscripts must include a data availability statement. This statement should provide the following information, where applicable:
- Accession codes, unique identifiers, or web links for publicly available datasets
- A description of any restrictions on data availability
- For clinical datasets or third party data, please ensure that the statement adheres to our policy

Available upon a reasonable request to the corresponding author. Note that sharing of pseudonymized personal data requires a data processor agreement (DPA) under the EU and Swedish laws.

Human research participants

Policy information about studies involving human research participants and Sex and Gender in Research.

Reporting on sex and gender	Sex information were obtained by self-report (of infants' parents). All findings from our analyses apply to both sexes. Consent was obtained from parent(s) of each participating infant for reporting of this information.<br>The overall number of each sex included from each study:<br>EASE: Female = 38 (52.1%), Male = 35 (47.9%)<br>BATSS: Female = 222 (49.1%), Male = 230 (50.9%)
Population characteristics	See below
Recruitment	See below
Ethics oversight	Both studies (EASE & BATSS) were approved by the Stockholm Regional Ethics Board (Stockholm Regionala Etikprövningsmyndigheten)

Note that full information on the approval of the study protocol must also be provided in the manuscript.

Field-specific reporting

Please select the one below that is the best fit for your research. If you are not sure, read the appropriate sections before making your selection.

☐ Life sciences ☒ Behavioural & social sciences ☐ Ecological, evolutionary & environmental sciences

For a reference copy of the document with all sections, see nature.com/documents/nr-reporting-summary-flat.pdf

Behavioural & social sciences study design

All studies must disclose on these points even when the disclosure is negative.

Study description	Prospective study of infants at elevated likelihood for autism (EASE cohort) and longitudinal infant twin study (BATSS)
Research sample	In both studies (EASE & BATSS), the participants were 5-month old infants (at first assessment) recruited from the greater Stockholm region
Sampling strategy	EASE: Infants at elevated risk of Autism as determined by the presence of direct family member(s), e.g. an older sibling, who was diagnosed with ASD (the case group) and infants who did not have any direct family member diagnosed with ASD (the control group). Those in the case group were recruited via paediatric clinics around greater Stockholm, while those in control group were recruited via the Swedish Population Registry.<br>BATSS: Same-sex infant twins from the general population, recruited via the Swedish Population Registry.
Data collection	EASE & BATSS: EEG data recording was done by trained research assistants using 128-channel EEG instruments (sensor & amplifier) with cap size appropriate for participants' age.<br>EASE: ADOS data was collected by in-person visits at 24 and 36 months and administered by trained clinicians. None of the clinicians was aware of the result of the EEG assessment.<br>BATSS: Behavioural assessments (ITC and QCHAT) data was collected by phone interviews with the parents at 14 months (ITC) and 24 months (QCHAT), respectively.
Timing	EASE: Data collection was done from 2013 to 2018<br>BATSS: Data collection was done from 2016 to 2020
Data exclusions	EASE: 18 infants excluded due to not having enough/bad EEG data<br>BATSS: 23 infants excluded from the beginning due to specific medical conditions (i.e. twin-to-twin transfusion syndrome, low birth weight, spina bifida, and seizure at birth), 112 excluded due to not having enough/bad EEG data; for Twin Modelling and GEE: 86 excluded due to incomplete pairs<br><br>* note: infants can be both (data) excluded and non-participating
Non-participation	EASE: 1 infant did not participate in EEG session, 8 did not participate in ADOS session at 24 months, and 20 did not participate in ADOS session at 36 months<br>BATSS: 35 did not participate in EEG session, 134 did not contribute to the ITC questionnaire at 14 months, and 211 did not contribute to the QCHAT questionnaire at 24 months<br><br>* note: infants can be both (data) excluded and non-participating<br>+ example: in BATSS, out of the 134 who did not contribute to ITC at 14 mo., some of them were also excluded due to bad EEG data

Randomization	not applicable; observational study

Reporting for specific materials, systems and methods

We require information from authors about some types of materials, experimental systems and methods used in many studies. Here, indicate whether each material, system or method listed is relevant to your study. If you are not sure if a list item applies to your research, read the appropriate section before selecting a response.

Materials & experimental systems

n/a	Involved in the study
☒	☐ Antibodies
☒	☐ Eukaryotic cell lines
☒	☐ Palaeontology and archaeology
☒	☐ Animals and other organisms
☒	☐ Clinical data
☒	☐ Dual use research of concern

Methods

n/a	Involved in the study
☒	☐ ChIP-seq
☒	☐ Flow cytometry
☒	☐ MRI-based neuroimaging
